# Clinical Surrogate Endpoints in Malattia Leventinese/Doyne Honeycomb Retinal Dystrophy: Findings From a Two-Year Natural History Study

**DOI:** 10.1167/iovs.67.11.24

**Published:** 2026-09-14

**Authors:** Clara Ehrenzeller, Giuseppe Cancian, Vasil Kostin, Arianna Peyla, Georg Ansari, Arianna Paris, Gabriela Grimaldi, Maximilian Pfau, Moreno Menghini

**Affiliations:** 1 Department of Ophthalmology, Ospedale Regionale di Lugano, Ente Ospedaliero Cantonale (EOC), Lugano, Switzerland; 2 Nuffield Laboratory of Ophthalmology, Nuffield Department of Clinical Neurosciences, University of Oxford, United Kingdom; 3 Department of Ophthalmology, University Hospital Basel, Basel, Switzerland; 4 Department of Ophthalmology, Guy's and St. Thomas’ NHS Foundation Trust, London, United Kingdom; 5 Department of Ophthalmology, University Hospital Bonn, Bonn, Germany

**Keywords:** inherited retinal disorder, Malattia Leventinese, Doyne Honeycomb Retinal Dystrophy, outcome measure, microperimetry

## Abstract

**Purpose:**

Malattia Leventinese/Doyne Honeycomb Retinal Dystrophy is a rare, inherited retinal dystrophy characterized by drusen formation and early vision loss. Its phenotypical similarities with dry age-related macular degeneration (AMD) make Malattia Leventinese a valuable monogenetic disease model for AMD. Identifying sensitive outcome measures is crucial for clinical trials. This study quantifies retinal pigment epithelium and drusen complex (RPEDC) increase and retinal layer degeneration using fully automated segmentation for multimodal imaging analysis, and investigates structural thresholds associated with functional decline through pointwise correlation.

**Methods:**

This multicenter cohort study included 25 patients (15 women and 10 men; median age = 60 years, interquartile range [IQR] = 51–73 years) with genetically confirmed Malattia Leventinese. Participants underwent multimodal imaging, including spectral-domain optical coherence tomography (SD-OCT), ultrawide fundus retinography and fundus autofluorescence (FAF), and mesopic and two-color scotopic microperimetry. Changes in retinal layer thicknesses and retinal sensitivity were assessed over 2 years using linear mixed-effects models, and structure–function breakpoints were estimated with piecewise models.

**Results:**

RPEDC thickness increased (+2.24 µm/y), whereas photoreceptor inner segments (−0.70 µm/y) and outer nuclear layer (−1.95 µm/y) thicknesses, in addition to choroidal thickness (−10.83 µm/y), decreased with disease progression. Total retinal thickness remained stable. Scotopic sensitivity declined over time, with −0.40 decibel (dB)/year at 505 nm and −0.37 dB/year at 627 nm, whereas mesopic sensitivity remained stable. Function decreased most in early disease stages, with the onset of drusen accumulation.

**Conclusions:**

In Malattia Leventinese, the total retinal thickness remains unchanged because the RPEDC increase offsets the thinning of outer retinal layers. Only scotopic function decreases significantly with progression and before the age of 55 years. Mesopic function may serve to track later stages. These findings support the outer nuclear layer and photoreceptor inner segment thicknesses as key structural endpoints and scotopic sensitivity as an early functional outcome measure for monitoring disease progression in ongoing and future clinical trials.

Malattia Leventinese, also known as Doyne Honeycomb Retinal Dystrophy, is a rare autosomal dominant inherited retinal disorder characterized by progressive vision loss typically beginning in the third to fourth decade of life and leading to severe visual impairment by the eighth or ninth decade, although onset of symptoms and visual impairment have shown to be variable.^1^ Malattia Leventinese is caused by a c.1033C > T (p.R345W) mutation in the *EFEMP1* gene,^2^ which results in the accumulation of misfolded fibulin-3 beneath the retinal pigment epithelium (RPE)^3^^,^^4^ and in complement activation, including complement factor B (CFB),^5^ contributing to the formation of drusen. Drusen initially appear in a radial, honeycomb pattern around the macula and optic disc, later coalescing and leading to RPE and outer retinal atrophy.^6^ In advanced stages, macular neovascularization may develop.^6^^,^^7^ It has been shown that the photoreceptor-RPE-choroid neurovascular unit alters faster in R345W Efemp1 mutated mice than in age-matched controls.^8^^,^^9^

Although rare, Malattia Leventinese shares striking phenotypic similarities with age-related macular degeneration (AMD), a leading cause of legal blindness worldwide.^3^^,^^4^^,^^6^^,^^10^^,^^11^ As such, Malattia Leventinese represents a valuable monogenic disease model to study disease mechanisms and potential therapies relevant to AMD.^4^^,^^11^ To date, no treatment has demonstrated a clinically meaningful benefit in visual function for either dry AMD or Malattia Leventinese.^12^ Recently approved complement inhibitors (e.g., C3 or C5 inhibitors) have shown efficacy in slowing the anatomic progression of RPE and outer retinal atrophy, but without a proven improvement in visual outcomes.^13^ Currently, a CFB inhibitor is being evaluated in an ongoing phase II trial in patients with AMD.^14^ Given that the deletion of the *CFB* gene in the *EFEMP1* knock-in mouse model reduces drusen development,^13^ drug repurposing for treating Malattia Leventinese warrants consideration in the near future.

To evaluate therapy effectiveness, reliable markers demonstrating disease progression are needed. We previously proposed to use RPE and drusen complex (RPEDC) thickness as a structural outcome measure in Malattia Leventinese, because, in a cross-sectional study, we demonstrated a direct relationship showing that increasing the RPEDC is associated with worsening mesopic, cone-, and rod-mediated sensitivity.^15^ However, to make it a reliable and meaningful outcome marker, confirmation through longitudinal assessment of function is required. With drusen accumulation, the interchange of nutrients and waste products between the RPE and the underlying choroid is impaired.^16^ Consequently, RPE cells become atrophic and lose their function.^17^ As established in other retinal diseases, functional testing with mesopic and two-color scotopic microperimetry offers precise assessment of retinal sensitivity from early to late stages,^18^^,^^19^ differentiating cone and rod function.^20^ Thus, microperimetry may serve as a sensitive endpoint to understand disease progression and monitor therapeutic efficacy in Malattia Leventinese, too.^18^^,^^21^^,^^22^ Besides validating RPEDC thickness as outcome measure, we need to explore other morphological markers demonstrating disease progression, using multimodal imaging. Artificial intelligence (AI) algorithms facilitate automatic image processing.^23^ Moreover, choroidal thickness has gained interest in AMD research,^24^^,^^25^ but remains largely unexplored in Malattia Leventinese.

In this natural history study, we aimed to quantify RPEDC increase and retinal layer degeneration using multimodal imaging analysis with fully automated segmentation. We further aimed to investigate structural thresholds associated with retinal function decline applying a pointwise correlation.

## Methods

### Study Design and Participants

This prospective, multicenter longitudinal cohort study was conducted at the Department of Ophthalmology, Ospedale Regionale di Lugano and the University Hospital Basel, Switzerland. Participants were recruited between 2021 and 2024 and underwent baseline, 1-year and 2-year follow-up examinations through 2025. The study protocol adhered to the tenets of the Declaration of Helsinki and was approved by the local ethics committee of Canton Ticino (CE 4421; BASEC 2023-01353). Written informed consent was obtained from all participants.

Patients with suspected Malattia Leventinese and their family members were screened. For the present analysis, inclusion required genetically confirmed Malattia Leventinese and availability of gradable baseline retinal imaging.

Disease severity was classified according to the previously described morphologic phenotype.^2^ Mild disease was defined by thickening of the Bruch's membrane-RPE complex with isolated drusen. Moderate disease was defined by numerous, partially confluent drusen with speckled hypo- and hyper-autofluorescence on fundus autofluorescence (FAF) imaging and focal RPE atrophy. Severe disease was defined by widespread RPE atrophy and diffuse confluent drusen accumulation.^2^

### Retinal Imaging

All participants underwent comprehensive multimodal retinal imaging. Spectral-domain optical coherence tomography (SD-OCT), with and without enhanced depth imaging (EDI), was acquired using the Heidelberg Spectralis (Heidelberg Engineering, Heidelberg, Germany) with a scan area of 5.9 × 1.9 mm centered on the fovea and B-scan spacing of 123 µm. Follow-up mode was used to ensure consistent scan placement across visits. Scans with insufficient image quality or segmentation failure were excluded from the corresponding analysis.

Ultrawide-field color fundus photography was obtained using the Optos imaging system (Optos PLC, Dunfermline, UK). FAF imaging was also performed as part of the multimodal imaging protocol.

### Functional Testing

Mesopic and two-color scotopic retinal sensitivities were measured using fundus-controlled perimetry (Macular Integrity Assessment [S-MAIA]; CenterVue, Padua, Italy), as previously described.^15^

Mesopic testing was performed under light-adapted conditions using the preset 68-point grid (10-2 pattern) with Goldmann III white stimuli. Background luminance was 1.27 cd/m^2^, stimulus duration was 200 ms, and the threshold strategy was 4-2 (sensitivity range = 0–36 decibel [dB]; maximum stimulus intensity = 318 cd/m^2^).

Scotopic microperimetry was conducted after 20 minutes of dark adaptation. Stimuli at 505 nm (cyan stimuli) were presented first, followed by stimuli at 627 nm (red stimuli), using a built-in 37-point grid covering the central 10 degrees. Background luminance was <0.0001 cd/m^2^, stimulus duration was 200 ms, and the threshold strategy was 4-2 (sensitivity range = 0–36 dB; maximum stimulus intensity = 2.54 scot cd/m^2^).

### Image Processing

**Figure 1.**
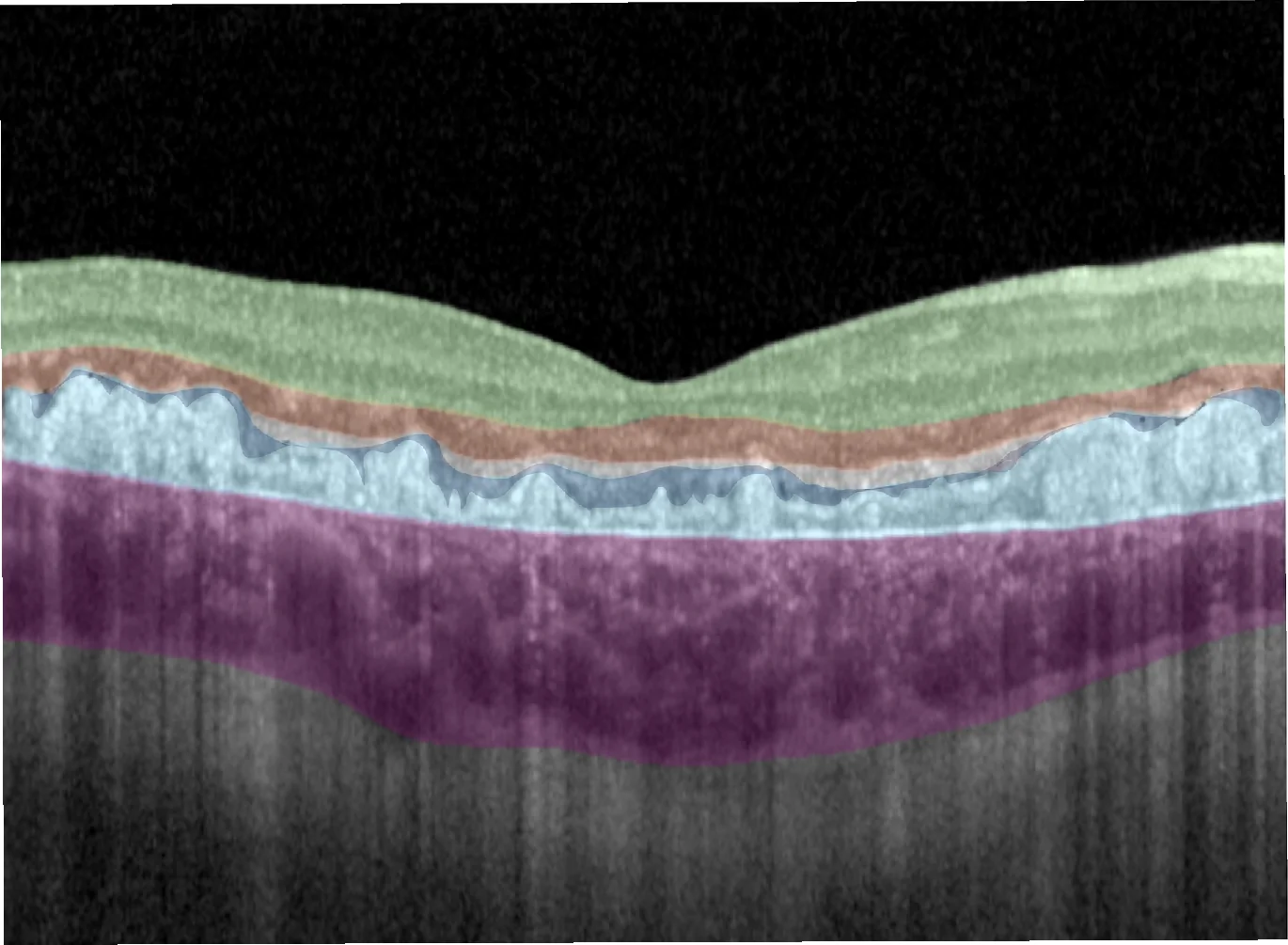
**Output of the fully automated B-scan segmentation.** Segmented retinal layers in a representative B-scan are color-coded as follows: choroid (*pink*), RPEDC (*blue*), photoreceptor outer segments (*purple*), photoreceptor inner segments (*white*), outer nuclear layer (*red*), and inner retinal layers (*green*).

Retinal layer segmentation from SD-OCT volume scans was performed using a previously validated convolutional neural network.^26^ The segmented boundaries were used to derive thickness maps for the inner retinal layers, outer nuclear layer, photoreceptor inner segments, photoreceptor outer segments, RPEDC, and choroid (Fig. 1).

After B-scan segmentation, en face thickness maps were generated for each layer. The Early Treatment Diabetic Retinopathy Study (ETDRS) grid was centered on the macula using inner retinal thickness as the anatomic reference. Thickness values were extracted from the central 3.46-degree diameter subfield corresponding to 1-mm diameter in an emmetropic eye and from the inner temporal, superior, nasal, and inferior subfields within the annulus spanning 1.73 to 5.19 degrees radius, corresponding to 0.5 to 1.5 mm in an emmetropic eye from the fovea. Thickness measurements were calculated for individual B-scans and subsequently averaged across all B-scans within an ETDRS subfield.

Automated segmentation previously demonstrated good agreement with manual grading (Dice coefficient = 0.82, 95% confidence interval [CI] = 0.80–0.85).^26^ Image processing was performed using Jupyter Notebook version 7.2.2 (Project Jupyter, Berkeley, CA, USA) and Fiji version 2.16.0 (ImageJ Community, Dresden, Germany).

To compare retinal sensitivity with retinal layer thickness on a point-by-point basis, each microperimetry examination was coregistered to the corresponding SD-OCT using an in-house deep learning image-matching pipeline. The MAIA infrared fundus image was aligned to the SD-OCT infrared localizer image, and a projective transformation (3 × 3 homography) was estimated from image correspondences using random sample consensus (RANSAC). This transformation mapped each microperimetry stimulus location onto the SD-OCT, enabling retinal layer thickness measurements to be sampled at the exact stimulus coordinates. Coregistration accuracy was verified by manual inspection of animated image overlays; inadequate registrations were repeated up to three times.

### Outcome Measures

Structural outcomes included the thickness of individual retinal layers. RPEDC thickness was defined as the thickness of the segmented RPEDC, extending from Bruch's membrane to the outer border of the RPEDC.

Functional outcomes included mesopic retinal sensitivity (MRS), scotopic retinal sensitivities at 505 nm (cyan) and 627 nm (red), and the scotopic wavelength difference within the central 10 degrees centered on the preferred retinal locus (PRL). The scotopic wavelength difference was calculated as the difference between the sensitivity at 627 nm and 505 nm. Test locations with 0 dB sensitivity at both wavelengths were excluded.

### Statistical Analysis

Statistical analyses were performed using RStudio (version 2024.04.2+764; Posit, PBC, Boston, MA, USA) with lme4 (1.1–37) and lmerTest (3.1–3). Data distribution was assessed using the Shapiro–Wilk test. Normally distributed variables are reported as mean ± standard deviation (SD), and non-normally distributed variables as median (interquartile range [IQR]). Statistical significance was defined as *P* < 0.05.

Descriptive statistics were calculated for structural and functional outcomes. Bonferroni correction was applied within hypothesis families: α = 0.01 for retinal layer comparisons (6 tests), α = 0.01 for ETDRS segments (4 comparisons), and α = 0.02 for functional outcomes (3 tests). No correction was applied to total retinal thickness per ETDRS subfield.

Longitudinal changes in retinal layer thickness and functional measures were evaluated using linear mixed-effects models, with time from baseline, expressed in years, as the fixed effect. Random intercepts for patient and for eye nested within patient were included to account for inter-eye correlation and repeated measurements over time.

Average layer thickness across ETDRS subfields was calculated by weighting values according to subfield area: ((π × 1.5^2^ − π × 0.5^2^)/4 × subfield thickness)/(π × 1.5^2^) for the four inner subfields and (π × 0.5^2^ × subfield thickness)/(π × 1.5^2^) for the central subfield.

Spearman correlations (ρ) between retinal layer thicknesses (individual layers and total retinal thickness) of ETDRS subfields and mean functional measures were computed at each visit. Correlations were Fisher z-transformed and regressed on visit (weights = *n* − 3) to estimate baseline correlation and longitudinal change.

To identify retinal layer thickness breakpoints associated with functional decline, pointwise structure–function relationships were modeled using broken-stick linear mixed-effects models. Retinal sensitivity was assumed to remain at a ceiling until thickness crossed a breakpoint, after which sensitivity declined linearly. This was implemented using a piecewise predictor, maximum (0, breakpoint − thickness), yielding a ceiling sensitivity and post-breakpoint slope. Breakpoints were estimated by profile likelihood. Models included age as a covariate and nested random intercepts for patient, eye, and visit. Separate models were fitted for each retinal layer and functional modality using responsive points (≥2 dB). Breakpoint 95% CIs and fitted-curve confidence bands were derived by patient-level cluster bootstrap (*N* = 1000). Broken-stick and linear mixed-effects models were compared using Akaike information criterion (AIC).

Segmented (piecewise linear) regression was used to identify age thresholds associated with functional decline. Model fit was compared using the AIC and Bayesian information criterion (BIC).

ChatGPT (version 5; OpenAI) was used to assist with routine programming in R software for data handling.

## Results

### Participant Characteristics

Thirty participants were enrolled. Three individuals without the EFEMP1 p.R345W mutation and two genetically confirmed patients who were unable to complete study procedures due to poor general health were excluded. Therefore, 25 patients (50 eyes) were included in the analysis (Table 1). Baseline SD-OCT imaging was available for all 25 participants, and microperimetry data were available for 22 participants.

**Table 1.** Demographics

	Patients With Baseline Visit	Patients With Follow-Up Visit After 1 Y	Patients With Follow-Up Visit After 2 Y
Patients, *n*	25	15	8
F sex, *n* [%]	15 [60]	9 [60]	6 [75]
Age, y, median [IQR]	56 [50–73]	53 [51–73]	63 [54–76]
Eyes, *n* [%]	50	26 [52]	20 [40]

Fifteen participants had at least one follow-up visit, and 8 participants completed the 2-year follow-up visit. Thus, 15 patients contributed to the longitudinal analysis, whereas all 25 patients were included in cross-sectional analyses.

Median age was 60 years (IQR = 51–73 years), and 60% of participants were women (*n* = 15). Disease stage and phenotypes were heterogeneous across the cohort, although severity was symmetric between eyes within patients. Supplementary Figure S1 illustrates multimodal imaging of participants with distinct disease stages. One patient was excluded from pointwise analyses because no acceptable alignment could be achieved.

### Longitudinal Structural Changes

**Figure 2.**
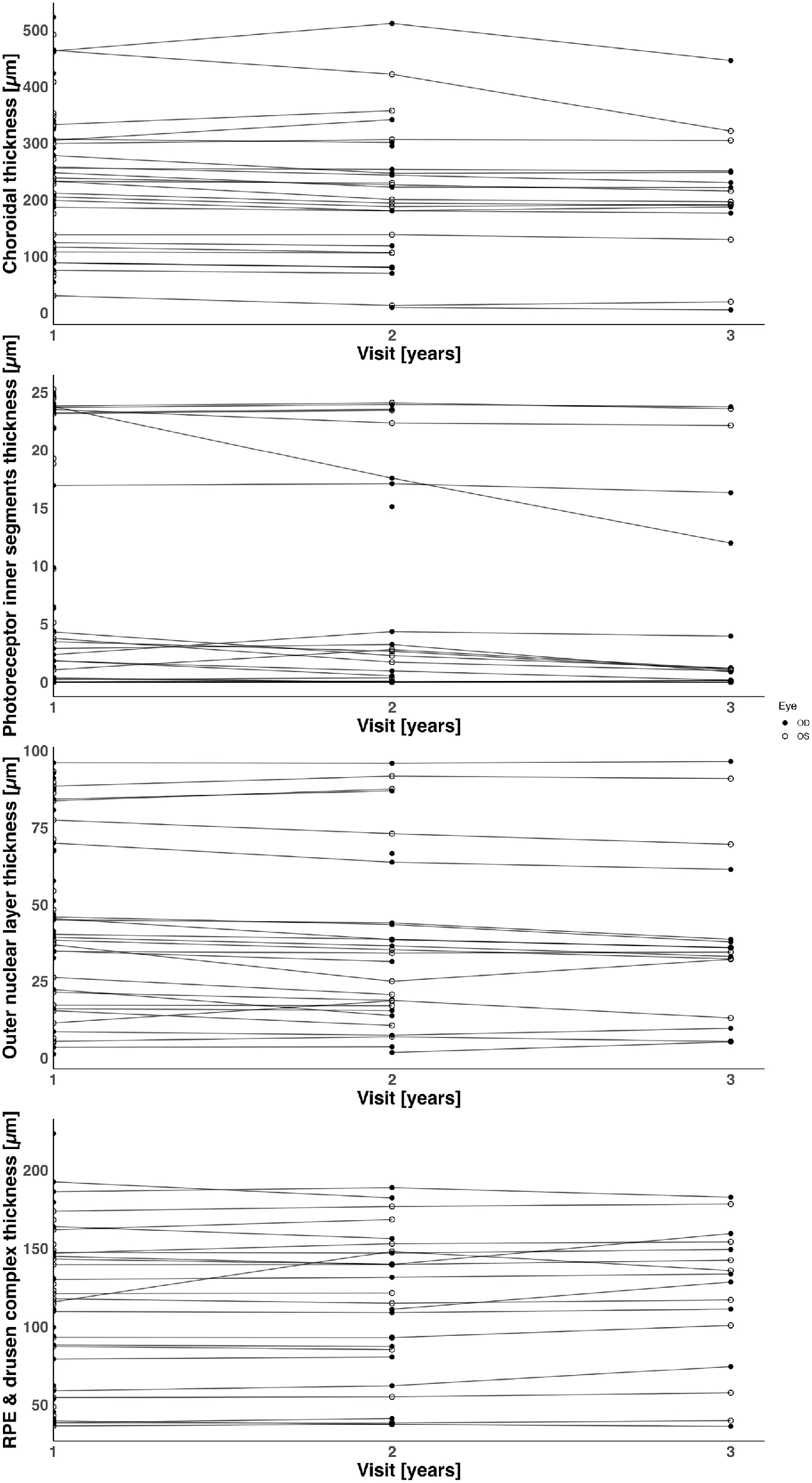
**Longitudinal changes in retinal layer thicknesses.** On these line plots, *dots* represent measurements of individual patients, whereas the *lines* connect the points of one patient over the follow-up visits. Each panel shows a different layer. *Closed dots* represent measurements from the right eye, whereas *open dots* represent the left eye.

Total retinal thickness, with or without inclusion of the choroid, remained stable over time. In contrast, several individual layers showed significant longitudinal changes (Table 2). Thickness of choroidal, photoreceptor inner segment, and outer nuclear layers decreased by −10.83 µm/year (*P* < 0.01), −0.70 µm/year (*P* < 0.01), and −1.95 µm/year (*P* < 0.001), respectively. In contrast, the RPEDC thickness increased in by +2.24 µm/year (*P* = 0.02) over time (Fig. 2). No significant longitudinal changes were observed in the remaining retinal layers. Thickness changes of the neurosensory retina and the RPEDC are summarized in Supplementary Table S1.

**Table 2.** Longitudinal Changes in Retinal Layer Thicknesses

	Inner Retinal Layers			Outer Nuclear Layer			Photoreceptor Inner Segments			Photoreceptor Outer Segments			RPE and Drusen Complex			Choroid		
Predictors	Estimates	CI	*P* Value	Estimates	CI	*P* Value	Estimates	CI	*P* Value	Estimates	CI	*P* Value	Estimates	CI	*P* Value	Estimates	CI	*P* Value
(Intercept)	165.62	156.00 to 175.24	**<0.001**	50.25	38.39 to 62.10	**<0.001**	11.10	7.04 to 15.16	**<0.001**	6.90	3.54 to 10.26	**<0.001**	102.82	83.80 to 121.83	**<0.001**	250.60	198.89 to 302.30	**<0.001**
Visit	1.52	−0.10 to 3.13	0.066	−1.95	−2.89 to −1.02	**<0.001**	−0.70	−1.16 to −0.24	**0.003**	0.47	−0.13 to 1.08	0.120	2.24	0.48 to 4.00	**0.013**	−10.83	−17.00 to −4.66	**0.001**
Random Effects																		
σ^2^	23.62			7.94			1.90			3.28			27.91			344.92		
τ_00_	248.04 _eye:X_			63.39 _eye:X_			11.01 _eye:X_			7.59 _eye:X_			547.14 _eye:X_			749.03 _eye:X_		
	418.34 _X_			842.67 _X_			95.35 _X_			61.78 _X_			1962.70 _X_			15910.68 _X_		
ICC	0.97			0.99			0.98			0.95			0.99			0.98		
N	2 _eye_			2 _eye_			2 _eye_			2 _eye_			2 _eye_			2 _eye_		
	25 _X_			25 _X_			25 _X_			25 _X_			25 _X_			25 _X_		
Observations	91			91			91			91			91			91		
Marginal *R*^2^/Conditional *R*^2^	0.002/0.966			0.002/0.991			0.003/0.982			0.002/0.955			0.001/0.989			0.004/0.980		

The *P* values in bold represent statistical significance.

In exploratory ETDRS subfield analyses of retinal thickness excluding the choroid, the central subfield showed the lowest mean thickness (274.92 µm). Longitudinally, nasal subfield thickness decreased by −1.72 µm/year, whereas the other inner ETDRS subfields showed small increases (Supplementary Table S2).

### Longitudinal Functional Changes

Scotopic retinal sensitivity declined significantly over time (Table 3). Sensitivity to 505-nm stimuli decreased by −0.40 dB/year (*P* < 0.001), and sensitivity to 627-nm stimuli decreased by −0.37 dB/year (*P* < 0.001). Mesopic retinal sensitivity and the scotopic wavelength difference did not show significant longitudinal change.

**Table 3.** Longitudinal Changes in Retinal Function

	MRS			Sensitivity at 505 nm			Sensitivity at 627 nm			Scotopic Wavelength Difference		
Predictors	Estimates	CI	*P* Value	Estimates	CI	*P* Value	Estimates	CI	*P* Value	Estimates	CI	*P* Value
(Intercept)	14.85	10.98 to 18.73	**<0.001**	10.15	7.16 to 13.14	**<0.001**	11.41	8.24 to 14.59	**<0.001**	−1.03	−1.88 to −0.19	**0.017**
Visit	−0.12	−0.36 to 0.12	0.317	−0.40	−0.60 to −0.20	**<0.001**	−0.37	−0.55 to −0.18	**<0.001**	−0.19	−0.31 to −0.07	**0.001**
Random Effects												
σ^2^	2.99			2.18			1.65			0.68		
τ_00_	13.22 _eye:X_			4.15 _eye:X_			5.38 _eye:X_			3.16 _eye:X_		
	70.36 _X_			43.50 _X_			48.97 _X_			1.94 _X_		
ICC	0.97			0.96			0.97			0.88		
N	2 _eye_			2 _eye_			2 _eye_			2 _eye_		
	20 _X_			20 _X_			20 _X_			20 _X_		
Observations	474			468			456			450		
Marginal *R*^2^/Conditional *R*^2^	0.000/0.965			0.002/0.956			0.001/0.971			0.004/0.882		

The *P* values in bold represent statistical significance.

### Structure–Function Associations

Correlations between retinal layer thickness and retinal sensitivity varied by layer and visit. Choroidal thickness demonstrated consistently strong positive correlations with all functional modalities across visits (ρ = 0.46–0.83). Outer retinal layers also showed strong structure–function associations, particularly for the outer nuclear layer (ρ = 0.34–0.84) and photoreceptor inner segments (ρ = 0.39–0.86), whereas photoreceptor outer segment correlations were strongest at baseline and weakened over follow-up (ρ = 0.30–0.83). In contrast, inner retinal layer thicknesses demonstrated weak or absent correlations with retinal sensitivity. The RPEDC showed inverse correlations with retinal function, strongest at baseline (ρ = −0.68 to −0.73), which attenuated over time. Across modalities, mesopic retinal sensitivity and scotopic sensitivity at 627 nm generally exhibited the strongest correlations with structural measurements. Detailed Spearman correlation coefficients are provided in Supplementary Table S3.

**Figure 3.**
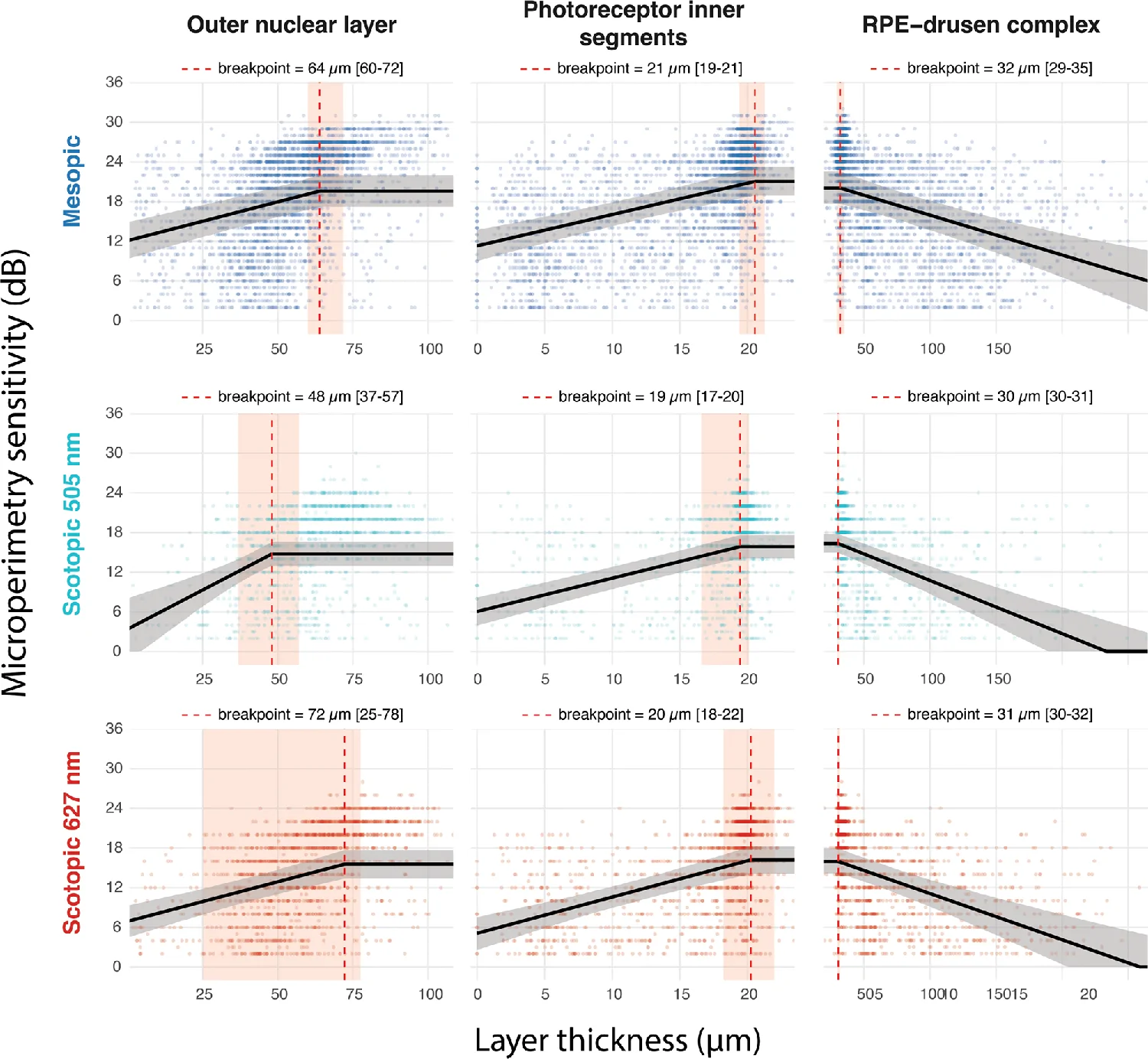
**Visual function loss in relation to retinal thickness change.** These panels illustrate mesopic sensitivity loss and scotopic sensitivity loss at 505 nm and 627 nm in relation to selected retinal layer thicknesses. *Colored* *dots* represent pointwise structure–function correlations. *Solid lines* indicate the linear mixed-effects model fit, with *shaded areas* representing the 95% confidence intervals. *Red dashed lines* indicate estimated breakpoints, with corresponding 95% confidence intervals shown in *red*.

Figure 3 demonstrated nonlinear structure–function relationships across retinal layers, with broadly similar breakpoints across mesopic and scotopic testing conditions. Retinal sensitivity remained relatively stable during early outer retinal thinning, followed by progressive decline beyond a structural breakpoint.

For the outer nuclear layer, breakpoints occurred at 64 µm for mesopic sensitivity, 48 µm for scotopic sensitivity at 505 nm, and 72 µm for scotopic sensitivity at 627 nm. Photoreceptor inner segment breakpoints occurred at approximately 20 µm across modalities, whereas photoreceptor outer segment breakpoints were consistently observed at 16 µm.

In contrast, increasing RPEDC thickness was associated with steadily declining retinal sensitivity, with breakpoints at approximately 30 to 32 µm. Choroidal associations were weaker and showed broad CIs. The CIs were narrowest for scotopic sensitivity at 505 nm and widest at 627 nm. Breakpoints for the choroid and all outer retinal layers are shown in Supplementary Figure S2 and summarized in Supplementary Table S4, together with the corresponding post-breakpoint slopes, age effects, and model comparison statistics.

Retinal sensitivity declined with age until approximately 55 years (*P* < 0.001) after which functional measures remained relatively stable. No age-related threshold was identified for RPEDC thickening.

A representative spatial structure–function analysis demonstrating the relationship between retinal sensitivity at 505 nm and outer retinal layer thicknesses is presented in Supplementary Figure S3.

## Discussion

This prospective longitudinal study characterized structural and functional progression in Malattia Leventinese over 2 years using multimodal retinal imaging and fundus-controlled perimetry. The main findings are: (1) over time, total retinal thickness remains stable; (2) outer nuclear layer and photoreceptor inner segment thicknesses are sensitive structural endpoints; and (3) scotopic sensitivity may serve as an early functional outcome measure. These findings suggest that layer-specific OCT metrics and scotopic microperimetry may be more sensitive than total retinal thickness for monitoring progression in Malattia Leventinese.

### Photoreceptor Degeneration

Over time, total retinal thickness remained stable, whereas several individual layers showed opposing trends, including thinning of the choroid, photoreceptor inner segments, and outer nuclear layer, and thickening of the RPEDC. The observed rate of choroidal thinning appeared greater than that reported for physiological aging, although this comparison should be interpreted cautiously because the present study did not include an age-matched control group.^27^ The concomitant thickening in RPEDC and thinning in layers of the outer retina explains the stability of total retinal thickness over time. This supports our conclusion that total retinal thickness is not suitable as a structural outcome measure in Malattia Leventinese.

These findings are consistent with progressive outer retinal degeneration in Malattia Leventinese. Accumulation of basal deposits and drusen at the RPE–Bruch's membrane interface^3^ may impair metabolic exchange among the choroid, RPE, and photoreceptors, ultimately contributing to photoreceptor loss.^16^^,^^28^ The concomitant choroidal thinning observed in this cohort may further reflect involvement of the outer retinal–choroidal complex. However, the present study was not designed to determine whether choroidal thinning is a cause or consequence of outer retinal degeneration.

### Functional Decline

Scotopic sensitivity declined significantly at both tested wavelengths, whereas mesopic sensitivity and the scotopic wavelength difference remained stable over follow-up.

Our study showed that patients with Malattia Leventinese have reduced mesopic and scotopic sensitivity compared with healthy subjects.^29^^,^^30^ Because 505-nm stimuli preferentially probe rod-mediated function and 627-nm stimuli reflect predominantly cone-mediated or mixed responses under scotopic conditions,^29^ the greater decline at 505 nm is consistent with early rod vulnerability.^20^ However, the stable scotopic wavelength difference suggests that rod- and cone-mediated responses may also decline in parallel over time, or that the study duration was insufficient to detect differential photoreceptor-specific change.^31^ Together, these findings suggest that scotopic sensitivity may serve as an early functional marker of disease progression, whereas mesopic sensitivity may reflect later-stage functional decline, a pattern that has also been proposed in AMD and other retinal dystrophies.^31^^,^^32^

### Structure—Function Relationships

Our findings suggest that functional decline in Malattia Leventinese is primarily driven by photoreceptor degeneration and drusen accumulation.

Retinal sensitivity remained relatively preserved during early outer retinal thinning, followed by progressive decline beyond a structural breakpoint, consistent with a period of functional reserve before overt photoreceptor decompensation. Photoreceptor outer segment thickness showed the strongest associations with retinal sensitivity across modalities, underscoring the importance of photoreceptor integrity for visual function,^15^ and suggesting that these OCT-derived measures may capture clinically meaningful tissue loss better than total retinal thickness. However, limited longitudinal outer segment degeneration may reduce its utility as a progression endpoint. In contrast, photoreceptor inner segment thinning may represent an early biomarker of disease progression, as functional decline occurred soon after measurable thinning developed.

Sensitivity loss occurred only after detectable outer retinal thinning, suggesting a therapeutic window in which structural changes precede substantial functional impairment. Consistent with prior AMD studies,^26^^,^^33^^–^^35^ outer nuclear layer and photoreceptor inner segment thicknesses may therefore represent relevant surrogate endpoints in Malattia Leventinese.

Although retinal sensitivity declined progressively with increasing RPEDC thickness, RPEDC may be less suitable as a clinical trial endpoint because therapies would more likely target photoreceptor preservation than drusen burden directly. Associations with choroidal and inner retinal changes were comparatively weak, suggesting these alterations may occur secondary to the primary disease process.

Although mesopic sensitivity showed stronger structural correlations, rod-mediated scotopic dysfunction appeared to precede mesopic impairment. Clinically, this may explain the early difficulty patients report with reading and visual tasks under dim illumination. The apparent plateau in retinal sensitivity after approximately 55 years may indicate that the most dynamic functional decline occurs earlier in the disease course. If confirmed in larger cohorts, this could help inform the timing of future interventional trials.

Overall, although the changes in retinal layer thickness were statistically significant, their average magnitude over a clinical-trial-comparable period remained small and close to the measurement error of OCT. Overcoming this limitation may require one of two approaches: either supplementing structural endpoints with complementary functional or combined structure-function information, or improving the measurement itself through newer modalities such as high-resolution OCT,^36^ which offers a higher signal-to-noise ratio.

Although the applied classification system^15^ is limited by its subjective criteria, it remains useful for broadly characterizing structural and functional alterations across disease stages.

### Limitations

Several limitations should be considered. First, the moderate sample size limited our ability to account for potential modifiers of disease progression, such as lifestyle factors or sex.^37^^,^^38^ Second, not all participants completed follow-up visits, often due to advanced age, long travel distances, or work-related constraints. In addition, patients with severely impaired vision were unable to complete microperimetry. This limited characterization of late-stage functional decline. The observation period may also have been insufficient to capture long-term disease progression.

Furthermore, microperimetry grids were centered on the PRL according to the standard MAIA protocol. In advanced disease, superior displacement of the PRL may have resulted in a slight underestimation of central functional loss. Additionally, retinal layer thicknesses and ETDRS grid definitions were based on visual angle. Because axial length data were unavailable, no subject-specific magnification correction was applied. Although axial length has minimal effect on axial retinal thickness measurements obtained from individual B-scans,^39^ it influences transverse OCT scaling such that ETDRS regions correspond to different physical retinal areas in eyes with differing axial lengths, potentially confounding the reported thickness measurements.^40^

## Conclusions

In summary, Malattia Leventinese is characterized by progressive outer retinal degeneration and drusen accumulation, accompanied by early loss of rod-mediated scotopic sensitivity, particularly before 55 years of age. Photoreceptor inner segment thinning may represent the earliest structural marker of disease progression, whereas outer nuclear layer thickness appears most sensitive for identifying a potential therapeutic window and is already an established biomarker for AMD.^26^^,^^33^^–^^35^ Functionally, scotopic sensitivity may serve as an early endpoint, whereas mesopic function may serve to track later stages. Together, these findings support combined layer-specific OCT metrics and scotopic microperimetry as surrogate endpoints for future natural history studies and interventional trials in Malattia Leventinese.

## Supplementary Material

Supplement 1

## References

[1] Michaelides M, Jenkins SA, Brantley MA Jr, et al. Maculopathy due to the R345W substitution in fibulin-3: distinct clinical features, disease variability, and extent of retinal dysfunction. *Invest Ophthalmol Vis Sci*. 2006; 47(7): 3085–3097. 16799055 10.1167/iovs.05-1600

[2] Stone EM, Lotery AJ, Munier FL, et al. A single EFEMP1 mutation associated with both Malattia Leventinese and Doyne honeycomb retinal dystrophy. *Nat Genet*. 1999; 22(2): 199–202. 10369267 10.1038/9722

[3] Marmorstein LY, Munier FL, Arsenijevic Y, Schorderet DF, McLaughlin PJ, et al. Aberrant accumulation of EFEMP1 underlies drusen formation in Malattia Leventinese and age-related macular degeneration. *Proc Natl Acad Sci*. 2002; 99(20): 13067–13072. 12242346 10.1073/pnas.202491599 PMC130587

[4] Hulleman JD . Malattia Leventinese/Doyne Honeycomb retinal dystrophy: similarities to age-related macular degeneration and potential therapies. In: Bowes Rickman C, LaVail MM, Anderson RE, Grimm C, Hollyfield J, Ash J, editors. *Retinal Degenerative Diseases*. Cham, Switzerland: Springer International Publishing; 2016: 153–158. 10.1007/978-3-319-17121-0_21 26427406

[5] Garland DL, Fernandez-Godino R, Kaur I, et al. Mouse genetics and proteomic analyses demonstrate a critical role for complement in a model of DHRD/ML, an inherited macular degeneration. *Hum Mol Genet*. 2014; 23(1): 52–68. 23943789 10.1093/hmg/ddt395 PMC3857944

[6] Sohn EH, Wang K, Thompson S, et al. Comparison of drusen and modifying genes in autosomal dominant radial drusen and age-related macular degeneration. *Retina Phila Pa*. 2015; 35(1): 48–57. 10.1097/IAE.0000000000000263 PMC5513174 25077532

[7] Serra R, Coscas F, Messaoudi N, Srour M, Souied E. Choroidal neovascularization in Malattia Leventinese diagnosed using optical coherence tomography angiography. *Am J Ophthalmol*. 2017; 176: 108–117. 28088509 10.1016/j.ajo.2016.12.027

[8] Zawadzki RJ, Meleppat RK, Manna S, et al. Longitudinal monitoring of Photoreceptor- RPE-choroid neurovascular unit (PRC- NVU) morphology and function in the mouse model of Doyne honeycomb retinal dystrophy. *Invest Ophthalmol Vis Sci*. 2018; 59(9): 5822.

[9] Kothandath KK, Meleppat RK, Karlen S, et al. In vivo studies of changes in photoreceptor-RPE-choroid neurovascular unit (PRC-NVU) morphology and function in the mouse models of Doyne honeycomb retinal dystrophy and RPE mitochondrial dysfunction. *Invest Ophthalmol Vis Sci*. 2020; 61(7): 190.

[10] Fu L, Garland D, Yang Z, et al. The R345W mutation in EFEMP1 is pathogenic and causes AMD-like deposits in mice. *Hum Mol Genet*. 2007; 16(20): 2411–2422. 17666404 10.1093/hmg/ddm198

[11] Marmorstein L . Association of EFEMP1 with malattia leventinese and age-related macular degeneration: a mini-review. *Ophthalmic Genet*. 2004; 25(3): 219–226. 15512998 10.1080/13816810490498305

[12] Vienne-Jumeau A, Bousquet E, Behar-Cohen F. Complement system modulation in age-related macular degeneration: navigating failures, building future successes. *Curr Opin Immunol*. 2025; 96: 102616. 40729928 10.1016/j.coi.2025.102616

[13] Crowley MA, Garland DL, Sellner H, et al. Complement factor B is critical for sub-RPE deposit accumulation in a model of Doyne honeycomb retinal dystrophy with features of age-related macular degeneration. *Hum Mol Genet*. 2022; 32(2): 204–217. 10.1093/hmg/ddac187 PMC9840207 35943778

[14] ClinicalTrials.gov. A masked, placebo‑controlled study to assess Iptacopan in age‑related macular degeneration. Identifier NCT05230537. Last Update Posted July 3, 2025. Accessed July 7, 2026. https://clinicaltrials.gov/ct2/show/NCT05230537.

[15] Ehrenzeller C, Cancian G, Paris A, Grimaldi G, Pfau M, Menghini M. Drusen volume as clinical outcome measure in subjects with Malattia Leventinese. *Retina*. 2025; 45(6): 1192. 39841918 10.1097/IAE.0000000000004407

[16] Hussain AA, Lee Y. Extracellular matrix (ECM) aging in the retina: the role of matrix metalloproteinases (MMPs) in Bruch's membrane pathology and age-related macular degeneration (AMD). *Biomolecules*. 2025; 15(8): 1059. 40867504 10.3390/biom15081059 PMC12383675

[17] Hussain AA, Lee Y, Marshall J. Understanding the complexity of the matrix metalloproteinase system and its relevance to age-related diseases: age-related macular degeneration and Alzheimer's disease. *Prog Retin Eye Res*. 2020; 74: 100775. 31473329 10.1016/j.preteyeres.2019.100775

[18] Pfau M, Jolly JK, Wu Z, et al. Fundus-controlled perimetry (microperimetry): application as outcome measure in clinical trials. *Prog Retin Eye Res*. 2021; 82: 100907. 33022378 10.1016/j.preteyeres.2020.100907 PMC12872260

[19] Sadda SR, Chakravarthy U, Birch DG, Staurenghi G, Henry EC, Brittain C. Clinical endpoints for the study of geographic atrophy secondary to age-related macular degeneration. *Retina Phila Pa*. 2016; 36(10): 1806–1822. 10.1097/IAE.0000000000001283 PMC5384792 27652913

[20] Taylor LJ, Josan AS, Pfau M, Simunovic MP, Jolly JK. Scotopic microperimetry: evolution, applications and future directions. *Clin Exp Optom*. 2022; 105(8): 793–800. 35025727 10.1080/08164622.2021.2023477

[21] Jolly JK, Nanda A, Buckley TMW, Pfau M, Bridge H, MacLaren RE. Assessment of scotopic function in rod–cone inherited retinal degeneration with the scotopic macular integrity assessment. *Transl Vis Sci Technol*. 2023; 12(2): 10. 10.1167/tvst.12.2.10 PMC9919682 36749581

[22] Bagdonaite-Bejarano L, Hansen RM, Fulton AB. Microperimetry in three inherited retinal disorders. *Semin Ophthalmol*. 2019; 34(4): 334–339. 31146612 10.1080/08820538.2019.1622025

[23] Morelle O, Wintergerst M, Finger RP. Multimodale Bildgebung und -auswertung im Zeitalter von künstlicher Intelligenz. *Ophthalmol*. 2020; 117(10): 965–972. 10.1007/s00347-020-01210-6 32845382

[24] Krytkowska E, Grabowicz A, Mozolewska-Piotrowska K, Ulańczyk Z, Safranow K, Machalińska A. The impact of vascular risk factors on the thickness and volume of the choroid in AMD patients. *Sci Rep*. 2021; 11: 15106. 34302055 10.1038/s41598-021-94676-6 PMC8302717

[25] Krytkowska E, Olejnik-Wojciechowska J, Grabowicz A, Safranow K, Machalińska A. Association between subretinal drusenoid deposits and age-related macular degeneration in multimodal retinal imaging. *J Clin Med*. 2023; 12(24): 7728. 38137797 10.3390/jcm12247728 PMC10744131

[26] Pfau M, von der Emde L, de Sisternes L, et al. Progression of photoreceptor degeneration in geographic atrophy secondary to age-related macular degeneration. *JAMA Ophthalmol*. 2020; 138(10): 1026–1034. 32789526 10.1001/jamaophthalmol.2020.2914 PMC7426886

[27] Lin CY, Huang YL, Hsia WP, Wang Y, Chang CJ. Correlation of choroidal thickness with age in healthy subjects: automatic detection and segmentation using a deep learning model. *Int Ophthalmol*. 2022; 42(10): 3061–3070. 35381895 10.1007/s10792-022-02292-8

[28] Booij JC, Baas DC, Beisekeeva J, Gorgels TGMF, Bergen AAB. The dynamic nature of Bruch's membrane. *Prog Retin Eye Res*. 2010; 29(1): 1–18. 19747980 10.1016/j.preteyeres.2009.08.003

[29] Pfau M, Lindner M, Fleckenstein M, et al. Test-retest reliability of scotopic and mesopic fundus-controlled perimetry using a modified MAIA (Macular Integrity Assessment) in normal eyes. *Ophthalmologica*. 2017; 237(1): 42–54. 27997924 10.1159/000453079

[30] Adeyoju DAO, Josan AS, Taylor LJ, MacLaren RE. An analysis of scotopic microperimetry in healthy adults. *Transl Vis Sci Technol*. 2025; 14(6): 18. 10.1167/tvst.14.6.18 PMC12177773 40488699

[31] Kong X, Ibrahim-Ahmed M, Bittencourt MG, et al. Longitudinal changes in scotopic and mesopic macular function as assessed with microperimetry in patients with Stargardt disease: SMART Study Report No. 2. *Am J Ophthalmol*. 2022; 236: 32–44. 34695402 10.1016/j.ajo.2021.10.014 PMC8957498

[32] Nebbioso M, Barbato A, Pescosolido N. Scotopic microperimetry in the early diagnosis of age-related macular degeneration: preliminary study. *BioMed Res Int*. 2014; 2014: 671529. 25548774 10.1155/2014/671529 PMC4274674

[33] Schmidt-Erfurth U, Waldstein SM, Klimscha S, et al. Prediction of individual disease conversion in early AMD using artificial intelligence. *Invest Ophthalmol Vis Sci*. 2018; 59(8): 3199–3208. 29971444 10.1167/iovs.18-24106

[34] Zekavat SM, Sekimitsu S, Ye Y, et al. Photoreceptor layer thinning is an early biomarker for age-related macular degeneration: epidemiologic and genetic evidence from UK Biobank OCT data. *Ophthalmology*. 2022; 129(6): 694–707. 35149155 10.1016/j.ophtha.2022.02.001 PMC9134644

[35] Vogl WD, Bogunović H, Waldstein SM, Riedl S, Schmidt-Erfurth U. Spatio-temporal alterations in retinal and choroidal layers in the progression of age-related macular degeneration (AMD) in optical coherence tomography. *Sci Rep*. 2021; 11(1): 5743. 33707539 10.1038/s41598-021-85110-y PMC7952738

[36] Meinke J, Raming K, Kessler C, Holz FG, Pfau M, Pfau K. High-resolution optical coherence tomography correlates of Peau d'Orange in Pseudoxanthoma Elasticum. *JAMA Ophthalmol*. 2025; 143(12): 1025. 41196613 10.1001/jamaophthalmol.2025.4031 PMC12593678

[37] Becker S, L'Ecuyer Z, Jones BW, Zouache MA, McDonnell FS, Vinberg F. Modeling complex age-related eye disease. *Prog Retin Eye Res*. 2024; 100: 101247. 38365085 10.1016/j.preteyeres.2024.101247 PMC11268458

[38] Fonteh CN, Palestine AG, Wagner BD, et al. Sex differences in RANTES (CCL5) in patients with intermediate age-related macular degeneration (AMD) and controls with no AMD. *Transl Vis Sci Technol*. 2022; 11(2): 12. 10.1167/tvst.11.2.12 PMC8842629 35133404

[39] Salmon AE, Sajdak BS, Collery RF, Carroll J. Axial scaling of OCT retinal images is independent of axial length. *Invest Ophthalmol Vis Sci*. 2017; 58(8): 4430–4430.

[40] Odell D, Dubis AM, Lever JF, Stepien KE, Carroll J. Assessing errors inherent in OCT-derived macular thickness maps. *J Ophthalmol*. 2011; 2011: 692574. 21869920 10.1155/2011/692574 PMC3157761

