# Supplementary material for: Clinical Surrogate Endpoints in Malattia Leventinese/Doyne Honeycomb Retinal Dystrophy: Findings From a Two-Year Natural History Study
Source: Invest Ophthalmol Vis Sci. 2026 Sep 14;67(11):24. doi: 10.1167/iovs.67.11.24 (PMC13576988; doi:10.1167/iovs.67.11.24)
Supplement: Supplementary file 1 [file iovs-67-11-24_s001.docx]

**Supplementary online content**

**Supplement to:** C. Ehrenzeller et al. Clinical Surrogate Endpoints in Malattia Leventinese / Doyne Honeycomb Retinal Dystrophy: Findings from a Two-Year Natural History Study

Table of Contents

[eTable 1. Change in Retinal Thickness for Neurosensory Retina and RPEDC Across Visits.	2](#_Toc230212208)

[eTable 2. Change in Total Retinal Thickness per ETDRS Subfield Across Visits.	3](#_Toc230212209)

[eTable 3. Correlations Between Function and Retinal Layers Across Visits.	4](#_Toc230212210)

[eTable 4. Model Parameters for Pointwise Structure**—**Function Relationships.	5](#_Toc230212211)

[eFigure 1. Multimodal Imaging According to Disease Severity.	6](#_Toc230212212)

[eFigure 2. Retinal Function Loss Related to Retinal Thickness Change for Individual Layers.	7](#_Toc230212213)

# **eTable 1. Change in Retinal Thickness for Neurosensory Retina and RPEDC Across Visits.**

The neurosensory retina is defined as the thickness from the inner retinal layers to the outer photoreceptor segments. Estimates, confidence intervals (CI) and *P*-values are reported.

# **eTable 2. Change in Total Retinal Thickness per ETDRS Subfield Across Visits.**

The total retina thickness is the combined thickness of all retinal layers, without the choroid. Estimates, confidence intervals (CI) and *P*-values are reported.

# **eTable 3. Correlations Between Function and Retinal Layers Across Visits.**

Correlations were computed for mesopic retina sensitivity (MRS) and scotopic sensitivities at 505 nm and 627 nm, at the baseline visit (BL) and at follow-up visits after 1 year (FU1) and 2 years (FU2). The spearman correlation coefficient rho is shown. A *P* <0.05 is marked with a *.

# **eTable 4.** **Model Parameters for Pointwise Structure—Function Relationships.**

For each retinal layer and functional modality, the table reports the estimated breakpoint thickness, ceiling sensitivity above the breakpoint, post-breakpoint slope, age effect, and model comparison statistics. Ceiling represents the fitted sensitivity plateau prior to the breakpoint. Slope represents the rate of sensitivity change per µm beyond the breakpoint. Age reflects the cross-sectional age-at-visit effect (dB/year) rather than within-patient progression. ΔAIC was calculated as AIC(linear mixed-effects model) − AIC(broken-stick model), with positive values favouring the breakpoint model. Evidence for a breakpoint was classified as negligible (ΔAIC <2), moderate (ΔAIC 2–10), or strong (ΔAIC >10).

# **eFigure 1. Multimodal Imaging According to Disease Severity.**

The panels representatively illustrate fundus autofluorescence (blue light) and multicolour fundus images of ML patients in a mild, moderate and severe stages.

# **eFigure 2. Retinal Function Loss Related to Retinal Thickness Change for Individual Layers.**

These panels illustrate mesopic sensitivity loss and scotopic sensitivity loss at 505 nm and 627 nm in relation to retinal layer thicknesses. Coloured dots represent pointwise structure–function correlations. Solid lines indicate the linear mixed-effects model fit, with shaded areas representing the 95% confidence intervals. Red dashed lines indicate estimated breakpoints, with corresponding 95% confidence intervals shown in red.

**eFigure 3. Spatial Distribution of 505 nm Sensitivity Across Retinal Layers by Disease Severity.**

These panels show the spatial distribution of retinal sensitivity at 505 nm overlaid on outer retinal layer, photoreceptor inner and outer segment, and RPE-drusen complex thickness maps across disease severity stages. Heatmaps represent relative thickness increases (µm), with grey indicating 0 µm and warmer colours indicating increasing thickness.
